# Psychiatric morbidity in children with *KCNJ11* neonatal diabetes

**DOI:** 10.1111/dme.13135

**Published:** 2016-05-21

**Authors:** P. Bowman, E. Broadbridge, B. A. Knight, L. Pettit, S. E. Flanagan, M. Reville, J. Tonks, M. H. Shepherd, T. J. Ford, A. T. Hattersley

**Affiliations:** ^1^ NIHR Exeter Clinical Research FacilityUniversity of ExeterExeterUK; ^2^ Royal Devon and Exeter NHS Foundation TrustExeterUK; ^3^ Dame Hannah Rogers TrustNewton AbbotUK; ^4^ Department of PsychologyUniversity of ExeterExeterUK; ^5^ Institute of Biomedical and Clinical ScienceUniversity of Exeter Medical SchoolExeterUK; ^6^ Institute of Health ResearchUniversity of Exeter Medical SchoolExeterUK

## Abstract

**Aims:**

Mutations in the *KCNJ11* gene, which encodes the Kir6.2 subunit of the pancreatic K_ATP_ channel, cause neonatal diabetes. *KCNJ11* is also expressed in the brain, and ~ 20% of those affected have neurological features, which may include features suggestive of psychiatric disorder. No previous studies have systematically characterized the psychiatric morbidity in people with *KCNJ11* neonatal diabetes. We aimed to characterize the types of psychiatric disorders present in children with *KCNJ11* mutations, and explore their impact on families.

**Methods:**

The parents and teachers of 10 children with neonatal diabetes due to *KCNJ11* mutations completed the Strengths and Difficulties Questionnaire and the Development and Wellbeing Assessment. Strengths and Difficulties Questionnaire scores were compared with normative data. Diagnoses from the Development and Wellbeing Assessment were compared with known clinical diagnoses.

**Results:**

Strengths and Difficulties Questionnaire scores indicated high levels of psychopathology and impact. Psychiatric disorder(s) were present in all six children with the V59M or R201C mutation, and the presence of more than one psychiatric disorder was common. Only two children had received a formal clinical diagnosis, with a further one awaiting assessment, and the coexistence of more than one psychiatric disorder had been missed. Neurodevelopmental (attention deficit hyperactivity disorder and autism) and anxiety disorders predominated.

**Conclusions:**

Systematic assessment using standardized validated questionnaires reveals a range of psychiatric morbidity in children with *KCNJ11* neonatal diabetes. This is under‐recognized clinically and has a significant impact on affected children and their families. An integrated collaborative approach to clinical care is needed to manage the complex needs of people with *KCNJ11* neonatal diabetes.

## What's new?

- This is the first study to systematically assess psychiatric morbidity in people with *KCNJ11* mutations, using validated, standardized diagnostic tools.
- The data show that *KCNJ11* mutations, in addition to causing neonatal diabetes, also cause psychiatric disorders that are clinically unrecognized but have high impact on families.
- This research highlights the need for early assessment and an integrated and collaborative approach to clinical care in people with *KCNJ11* neonatal diabetes.

**What's new?**

- This is the first study to systematically assess psychiatric morbidity in people with *KCNJ11* mutations, using validated, standardized diagnostic tools.
- The data show that *KCNJ11* mutations, in addition to causing neonatal diabetes, also cause psychiatric disorders that are clinically unrecognized but have high impact on families.
- This research highlights the need for early assessment and an integrated and collaborative approach to clinical care in people with *KCNJ11* neonatal diabetes.

## Introduction

Mutations in *KCNJ11*, which encodes the Kir6.2 subunit of the K_ATP_ channel, are the commonest cause of neonatal diabetes. These are important to diagnose because > 90% of people with these mutations can transfer from insulin treatment to an oral sulfonylurea, achieving excellent glycaemic control 1. *KCNJ11* is expressed in the brain as well as the pancreas 2, explaining why ~ 20% of people with mutations in this gene have a neurological phenotype known as DEND (developmental delay, epilepsy and neonatal diabetes) syndrome 3. Even those without an overt neurological phenotype have recently been shown to have attention deficits and developmental coordination disorder on neuropsychological testing 4.

Case reports and animal data suggest that *KCNJ11* mutations may be associated with childhood psychiatric disorders. Two people with the commonest DEND mutation, V59M, have been reported to have attention deficit hyperactivity disorder (ADHD) 5. Mouse models with V59M mutations targeted to neuronal tissue have replicated the hyperactive phenotype, and show increased exploratory behaviour and reduced anxiety behaviour consistent with the inattention and impulsivity reported in humans, suggesting a role for *KCNJ11* in emotional regulation 6. Autism (comprising impaired language and social interaction and restricted/repetitive behaviours) has also been reported in one person with the V59M mutation 7. No previous studies have systematically assessed the psychiatric morbidity in people with *KCNJ11* neonatal diabetes, or the impact that this has on families.

We aimed to characterize the types of psychiatric disorders present in children and adolescents with *KCNJ11* mutations, and explore the impact of these on families.

## Methods

### Participants

We recruited 10 children from the UK (median age 8.5, range 6–17 years) with neonatal diabetes due to a mutation in the *KCNJ11* gene.

### Study procedures

#### Ethical approval

Ethical approval was obtained for the study from the National Research Ethics Service Committee South West–Exeter.

#### Recruitment and consent

Patients were recruited at a neonatal diabetes family event in Exeter. Valid informed consent was obtained from parents (at the family event) and teachers (at a later date).

#### Developmental and physical health history

Parents reported the ages at which their child achieved major fine and gross motor, social and speech and language milestones, their child's educational attainment, and any interventions required, for example, speech and language therapy, extra support at school. They also gave details of their child's physical and mental health history and current diabetes medication.

#### Psychiatric evaluation

Parents and teachers completed the Development and Wellbeing Assessment (DAWBA) and the Strengths and Difficulties Questionnaire (SDQ). The DAWBA is a standardized diagnostic interview that combines structured and semi‐structured approaches to generate Diagnostic and Statistical Manual of Mental Disorders IV (DSM–IV) 8 psychiatric diagnoses on 5–17‐year‐olds. Parallel versions exist for parents and young people aged 11–17 years, with a briefer questionnaire for teachers. It covers common emotional, behavioural and hyperactivity disorders, as well as less common but sometimes more severe psychiatric disorders. Clinicians assess the data from all available informants to assign diagnoses according to DSM–IV. The initial DAWBA validation study showed excellent discrimination between community and clinic samples in rates of diagnosed disorder 9. Since then, the DAWBA has been widely used in British national surveys and as a tool to aid clinical assessment in many countries.

The SDQ consists of 25 items relating to emotional symptoms, conduct problems, hyperactivity/inattention, peer relationships and prosocial behaviour. Scores across the first four subscales (5 items each) are summed to create a ‘total difficulties score' that ranges from 0 to 40. The impact supplement measures distress and social impairment caused by the child's difficulties. The reliability and validity of the SDQ make it a useful screen for psychopathology in children 10.

### Data analysis

The total difficulties, impact scores and psychiatric diagnoses were compared with normative data for ~ 8000 school‐age children from the British Child and Adolescent Mental Health Survey 2004 11.

## Results

### Clinical and developmental history

Parents reported high levels of developmental delay and learning difficulties (Table S1). Nine of ten children required intervention to assist education or development. Psychiatric morbidity (Table S1) was recognized: two children had a clinical diagnosis of autism, one was awaiting assessment due to probable autism and one had ‘autistic tendencies'. Another child required psychological support for low mood.

### SDQ

**Figure 1:**
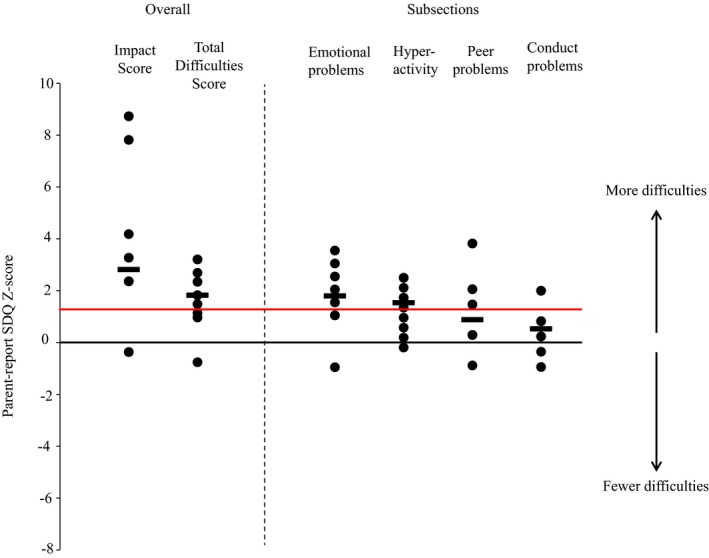
Patient difficulties as shown by parent‐report SDQ scores (presented as Z‐scores). Individuals are represented black circles and group medians as black bold horizontal lines. Zero on the *x*‐axis represents school‐age population mean, red horizontal line represents suggested clinical cut‐point (90th percentile).

Both parents (Fig. 1) and teachers (Fig. S1) reported high levels of psychopathology. Parent‐reported median impact and median total difficulties *Z*‐scores were 2.8 and 1.8 compared with the general school‐age population (*Z*‐score > 1.3 psychiatric evaluation suggested). Problems were most marked in emotional difficulties and hyperactivity. Prosocial behaviour scores were also reduced compared with the background population (median *Z*‐scores –1 and –0.7 for parent and teacher report, respectively).

### DAWBA

Clinical evaluation of parents' and teachers' responses to the DAWBA showed that definite psychiatric disorder(s) were present in six of ten children, but only two had a clinical diagnosis, with a further one awaiting formal assessment (Table 1). All children with either V59M (*n* = 4) or R201C (*n* = 2) had a definitive psychiatric diagnosis on the DAWBA. There was more than one disorder in four of six children, which was not recognized clinically. The prevalence of psychiatric disorder in British school children using the DAWBA is 10% 11, so the level of psychiatric morbidity is this cohort is higher than the background population (*P* = 0.0001 for a one‐sample proportion test).

**Table 1:** Clinical diagnoses and diagnoses obtained from DAWBA questionnaires

Case	Mutation	Clinical diagnoses	DAWBA diagnoses (DSM‐IV classification)		
			Neurodevelopmental disorders	Anxiety disorders	Behavioural disorders
1	V59M	–	Autism ADHD (combined)^a^	Other anxiety disorder	–
2	V59M	–	ADHD (combined)	–	–
3	V59M	Autism	Autism ADHD (combined)	Other anxiety disorder	–
4	V59M	Autism	Autism ADHD (combined)	Separation anxiety Specific phobia	–
5	R201C	–	–	Other anxiety disorder	–
6	R201C	–	Autism (probable)	Separation anxiety	Oppositional defiant disorder
7	K170R	–	–	–	
8	I182V	–	–	–	Other disruptive disorder (probable)
9	K170N	–	–	–	–
10	R201H	–	–	–	–

ADHD, attention deficit hyperactivity hisorder

a (combined) denotes all three features (hyperactivity, impulsivity and inattention) present.

Neurodevelopmental disorders were prominent (autism and ADHD). Three of ten children had both autism and ADHD; although DSM–IV criteria exclude ADHD as a diagnosis in the presence of autism, clinical practice and DSM–V have moved towards assigning both due to the high impact on families and need for clinical intervention. Anxiety disorders were common, with five of ten children being diagnosed with at least one anxiety disorder.

Three children had additional probable diagnoses, but we could not make definitive diagnoses based on the DAWBA. Case 6 (R201C) was assigned a probable diagnosis of autism (and is awaiting formal diagnostic assessment for autism by his local services), and Case 8 (I182V) had a probable diagnosis of other disruptive (conduct) disorder, but this related to behaviour more than 6 months previously which was now resolving. Finally, Case 9 (K170N) was supported by health psychology and a nurture group for low mood and self‐esteem, but did not reach the DAWBA diagnostic threshold for an emotional disorder.

## Discussion

Neurodevelopmental disorders (autism and ADHD) and/or anxiety disorders were present in all six children with sulfonylurea‐treated neonatal diabetes due to V59M or R201C *KCNJ11* mutations. Most of these psychiatric disorders had not been diagnosed in clinical practice.

Definitive psychiatric diagnosis occurring only in those with the V59M or R201C mutation is consistent with the previous literature. These are the two commonest mutations associated with neurological/developmental features, which are almost invariable in V59M and inconsistently reported in R201C 3. There is a clustering in the type of psychiatric disorder; ADHD is present in all four children with the V59M mutation, consistent with previous reports of ADHD in people and hyperactivity, inattention and impulsivity in mice with the mutation 2, 5, 6. Autism, found in four children, has previously been reported in a single patient 7. The presence of anxiety disorders in four children differs from the reduced anxiety behaviour noted in the V59M mouse model 6.

One of the most striking features of the assessment process was the impact on families of the difficulties identified. In those most severely impaired, parents had become full‐time carers for their children. Some families reported that their children needed more support, which suggests that awareness of the psychological problems faced by such families should be raised among healthcare professionals involved in their care. The complex pattern of needs that we identified requires a fully integrated and collaborative approach involving parents, carers, general practitioners, paediatric endocrinologists, occupational therapists, clinical/educational psychologists, teachers, special educational needs coordinators and child and adolescent mental health services.

## Limitations of the study

Because of the rarity of the condition, the number of participants recruited was small. In addition, the families who attended our family day may not be representative of all people with *KCNJ11* neonatal diabetes. Psychiatric difficulties could make patients more reluctant to attend a public meeting with considerable travelling or make them more likely to attend to seek advice. The total number of UK patients aged 5–17 years with *KCNJ11* mutations at the time of the study was 21, therefore 48% of those eligible for inclusion in the study took part. Furthermore, our cohort did have significantly more V59M and R201C mutations than in the total UK paediatric cohort (60% vs. 26% *P* = 0.049). For these reasons, we have been unable to use this study to provide prevalence data on the psychiatric features associated with *KCNJ11* mutations. However, our systematic and detailed assessments found considerable unrecognized psychiatric morbidity in this group.

## Further work

Our research suggests that psychiatric morbidity predominantly affects people with V59M and R201C mutations and most of these mutation carriers are affected. A larger study assessing more patients with these and other mutations will give information on prevalence and the extent to which psychiatric morbidity forms part of a specific phenotype/genotype relationship. In addition, further studies are needed to assess the effects of sulfonylurea therapy on psychiatric symptoms in people with *KCNJ11* mutations.

## Conclusions/implications

Systematic assessment using standardized validated questionnaires reveals a range of psychiatric morbidity in children with *KCNJ11* neonatal diabetes. This is under‐recognized clinically and has a significant impact on affected children and their families. An integrated and collaborative approach to clinical care is needed to ensure early identification and appropriate management of the complex needs of people with diabetes due to *KCNJ11* mutations.

## Funding sources

ATH is supported by a Wellcome Trust Senior Investigator award (Grant number 098395/Z/12/Z). SEF has a Sir Henry Dale Fellowship jointly funded by the Wellcome Trust and the Royal Society (Grant Number 105636/Z/14/Z). MHS and BAK are supported by the NIHR Exeter Clinical Research Facility.

## Competing interests

None declared.

## Author contributions

ATH, PB, TJF and JT contributed to conception and design of the study. PB, TJF, BK, LP, MS, JT and MR contributed to data acquisition. PB, TJF, ATH, SEF, MS and EB contributed to analysis and interpretation of data. PB, TJF and ATH drafted the article and all other authors critically revised it. All authors approved the final version prior to submission.

## Supporting information


**Table S1.** Patients characteristics and clinical information

**Figure S1.** Patient difficulties as shown by teacher‐report SDQ scores.

## References

[1] Pearson ER , Flechtner I , Njolstad PR , Malecki MT , Flanagan SE , Larkin B *et al* Switching from insulin to oral sulfonylureas in patients with diabetes due to Kir6.2 mutations. N Engl J Med 2006; 355: 467–477. 16885550 10.1056/NEJMoa061759

[2] Clark RH , McTaggart JS , Webster R , Mannikko R , Iberl M , Sim XL *et al* Muscle dysfunction caused by a KATP channel mutation in neonatal diabetes is neuronal in origin. Science 2010; 329: 458–461. 20595581 10.1126/science.1186146 PMC5890903

[3] Hattersley AT , Ashcroft FM . Activating mutations in Kir6.2 and neonatal diabetes: new clinical syndromes, new scientific insights, and new therapy. Diabetes 2005; 54: 2503–2513. 16123337 10.2337/diabetes.54.9.2503

[4] Busiah K , Drunat S , Vaivre‐Douret L , Bonnefond A , Simon A , Flechtner I *et al* Neuropsychological dysfunction and developmental defects associated with genetic changes in infants with neonatal diabetes mellitus: a prospective cohort study [corrected]. Lancet Diabetes Endocr 2013; 1: 199–207. 10.1016/S2213-8587(13)70059-7 24622368

[5] Sagen JV , Raeder H , Hathout E , Shehadeh N , Gudmundsson K , Baevre H *et al* Permanent neonatal diabetes due to mutations in KCNJ11 encoding Kir6.2: patient characteristics and initial response to sulfonylurea therapy. Diabetes 2004; 53: 2713–2718. 15448106 10.2337/diabetes.53.10.2713

[6] Lahmann C , Clark RH , Iberl M , Ashcroft FM . A mutation causing increased KATP channel activity leads to reduced anxiety in mice. Physiol Behav 2014; 129: 79–84. 24582665 10.1016/j.physbeh.2014.02.031 PMC5576528

[7] Tonini G , Bizzarri C , Bonfanti R , Vanelli M , Cerutti F , Faleschini E *et al* Sulfonylurea treatment outweighs insulin therapy in short‐term metabolic control of patients with permanent neonatal diabetes mellitus due to activating mutations of the *KCNJ11* (KIR6.2) gene. Diabetologia 2006; 49: 2210–2213. 16816952 10.1007/s00125-006-0329-x

[8] American Psychiatric Association (APA) . Diagnostic and Statistical Manual of Mental Disorders, 4th edn (DSM‐IV). Washington, DC: APA, 1994.

[9] Goodman R , Ford T , Richards H , Gatward R , Meltzer H . The Development and Well‐Being Assessment: description and initial validation of an integrated assessment of child and adolescent psychopathology. J Child Psych Psych 2000; 41: 645–655. 10946756

[10] Goodman R . Psychometric properties of the strengths and difficulties questionnaire. J Am Acad Child Psy 2001; 40: 1337–1345. 10.1097/00004583-200111000-00015 11699809

[11] Green HMA , Meltzer H , Ford T , Goodman R . Mental Health of Children and Young People in Great Britain, 2004. Basingstoke: Palgrave Macmillan, 2005.

