# Supplementary material for: Psychiatric morbidity in children with KCNJ11 neonatal diabetes
Source: Diabet Med. 2016 May 21;33(10):1387–91. doi: 10.1111/dme.13135 (PMC5031218; doi:10.1111/dme.13135)
Supplement: Supplementary file 1 — Table S1. Patients characteristics and clinical information [file DME-33-1387-s001.docx]

**Table S1** Patient characteristics and clinical information

| Case | Age (yrs) | Sex | Mutation (Gene) | Parental Report of Developmental Milestones (Interventions) | Psychiatric history / epilepsy / other relevant clinical history | Current diabetes treatment (dosage in mg/kg/day^a^) | HbA1c ^b^ IFCC mmols/mol (DCCT %) | Age at diagnosis of diabetes (weeks) | Age of transfer to sulphonylureas (if applicable) |
| --- | --- | --- | --- | --- | --- | --- | --- | --- | --- |
| 1 | 14 | M | V59M (*KCNJ11*) | Delayed – current mental age 4 (attends Special School) | ‘Some autistic tendencies’ noted as a younger child. Epilepsy diagnosed aged 10 years; treatment Epilim (sodium valproate) 400mg BD. | Glibenclamide 10mg TDS (0.7mg/kg/day) | 36 (5.4) | 1 | 4 years |
| 2 | 6 | M | V59M (*KCNJ11*) | Delayed – current mental age 3 (1:1 support at school, SEN^d^ statement) | Younger brother of case 3. | Glibenclamide 10mg BD (1mg/kg/day) | 30 (4.9) | 9 | On SU since diagnosis |
| 3 | 17 | M | V59M (*KCNJ11*) | Delayed – current mental age 3 (attends Special School) | Autism diagnosis. Under local ^g^CAMHS team. | Glibenclamide 30mg breakfast, 35mg dinner (1mg/kg/day) | 33 (5.2) | 1 | 10 years |
| 4 | 6 | F | V59M (*KCNJ11*) | Delayed – current mental age 3 (25 hours/week 1:1 support at school, SEN statement, uses Makaton to aid communication) | Autism diagnosis July 2014. Speech improved following recent increase in SU from 2.6mg / day. | Glibenclamide 11mg BD (1mg/kg/day) | 38 (5.6) | 12 | 12 months |
| 5 | 16 | F | R201C (*KCNJ11*) | Developmental concerns re: social interaction, learning difficulties at school (on enhanced learning programme – current mental age 12) | Difficulties with language expression / comprehension and mathematics. | Glibenclamide 20mg breakfast, 15mg lunch, 20mg dinner (1.1mg/kg/day) | 55 (7.2) | 11 | 10 years |
| 6 | 9 | M | R201C (*KCNJ11*) | Speech delay (speech and language therapy when younger, support at school from teaching assistant) | Probable autism – awaiting formal assessment. | Glibenclamide 4.2mg breakfast, 3mg lunch, 3.7mg dinner (0.3mg/kg/day) | 37 (5.5) | 1 | 4.5 months |
| 7 | 8 | M | K170R (*KCNJ11*) | Speech delay (speech & language therapy, 20 hours/week support from SENCO^f^, IEP^e^) | Being assessed for dyslexia (difficulties with maths/ literacy/ spellings /letter and shape formation). | Glibenclamide 5mg breakfast, 5mg lunch, 2.5mg dinner (0.48mg/kg/day) | 32 (5.1) | 33 | 15 months |
| 8 | 15 | F | I182V (*KCNJ11*) | Mild speech delay (some 1:1 support at school, particularly during examinations) | Diagnosed with ‘borderline’ dylsexia. Spent some time on gliclazide; found concentration improved when switched to glibenclamide. | Glibenclamide 15mg breakfast, 10mg dinner; noted to not be taking her medication regularly (0.5mg/kg/day) | 127 (13.8) | 1 | N/A^c^ (TNDM^h^); Insulin 8-9 months, no Rx then SU aged 11 years |
| 9 | 8 | M | K170N (*KCNJ11*) | Mild speech and motor delay (speech and language therapy as toddler) | Attends ‘nurture group’ at school to help confidence. Excels academically. Under paediatric diabetes psychology service due to low mood related to diabetes. | Glibenclamide 5mg breakfast, 2.5mg dinner (0.29mg/kg/day) | 33 (5.2) | 11 | 5 months |
| 10 | 6 | F | R201H (*KCNJ11*) | Normal | Nil | Glibenclamide 2.5mg TDS (0.4mg/kg/day) | 42 (6.0) | 12 | 12 months |

^a^Where no recent weight available, weight calculated based upon CDC paediatric growth charts using 50^th^ percentile (<http://www.cdc.gov/growthcharts> 2000) ^b^ Most recently available result ^c^N/A = Not applicable ^d^SEN = special educational needs ^e^IEP = Individualised education program ^f^SENCO = special educational needs coordinator ^g^CAMHS = child and adolescent mental health services ^h^TNDM = Transient neonatal diabetes mellitus.
