# Supplementary material for: Psychiatric morbidity in children with KCNJ11 neonatal diabetes
Source: Diabet Med. 2016 May 21;33(10):1387–91. doi: 10.1111/dme.13135 (PMC5031218; doi:10.1111/dme.13135)
Supplement: Supplementary file 2 — Figure S1. Patient difficulties as shown by teacher‐report SDQ scores. [file DME-33-1387-s002.pdf]

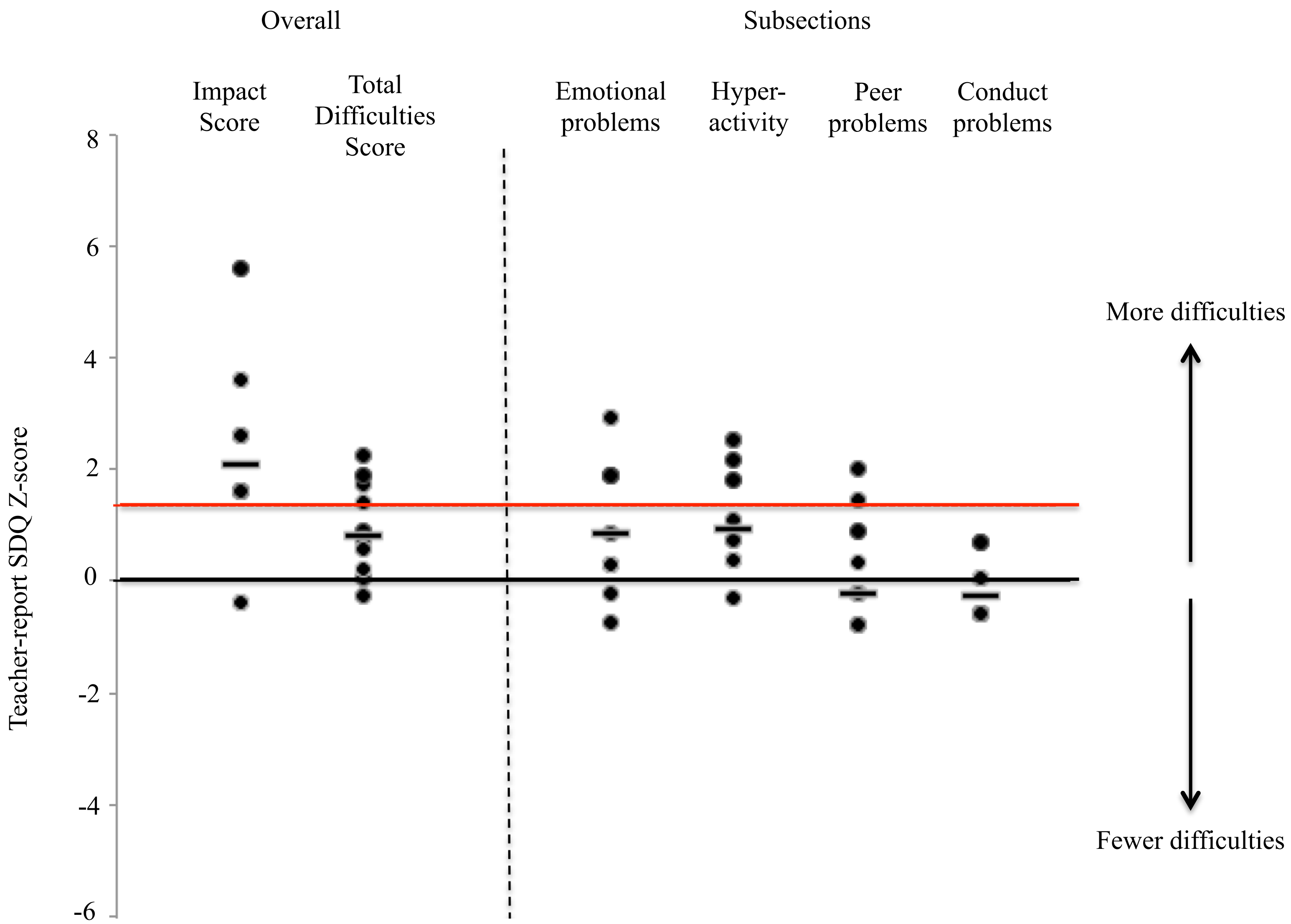

Figure S1. Patient difficulties as shown by teacher-report SDQ scores (presented as Z-scores). Individuals are represented as black circles and group medians as black bold horizontal lines. Zero on the x-axis represents school-age population mean, red horizontal line represents suggested clinical cut-point (90th percentile).
